# Efficacy and safety of sympathetic radiofrequency thermocoagulation in the treatment of cold hypersensitivity

**DOI:** 10.3389/fneur.2022.1026334

**Published:** 2022-10-24

**Authors:** Yuefeng Liao, Chi Xu, Jianmei Xia, Huadong Ni, Zhiqiang Zhang, Chunjue Ni

**Affiliations:** ^1^Department of Anesthesiology and Pain Research Center, The Affiliated Hospital of Jiaxing University, Jiaxing, China; ^2^Department of Anesthesiology, The First Affiliated Hospital of Wenzhou Medical University, Wenzhou, China

**Keywords:** cold hypersensitivity, sympathetic, radiofrequency thermocoagulation, efficacy, safety

## Abstract

**Background:**

Cold hypersensitivity (CH) is a sensation of cold in the limbs and (or) body of a patient in an environment that is not considered cold by unaffected people, or a strong feeling of cold at a relatively low temperature. However, the currently available treatments are limited and often unsatisfactory. This study aimed to evaluate the clinical efficacy and safety of the sympathetic radiofrequency thermocoagulation (RF-TC) technique in patients with CH disorder.

**Methods:**

The study is a retrospective analysis. A total of 71 were entered into the final analysis and all patients underwent computed tomography-guided thoracic (lumbar) sympathetic nerve RF-TC on an elective basis. The values of terminal temperature (T) and perfusion index (PI) of patients before and after treatment were recorded. Patients were followed up clinically at regular intervals and their Visual Analog Scale (VAS) and Pittsburgh Sleep Quality Index (PSQI) scores were recorded to detect postoperative complications and assess patient satisfaction with the treatment.

**Results:**

All patients completed the radiofrequency treatment. Compared with the preoperative period, VAS and PSQI scores were significantly lower at all postoperative time periods (*P* < 0.001). Patients had significantly higher postoperative terminal temperatures and perfusion indices on the right and left sides than before surgery (*P* < 0.001). The overall patient satisfaction score was 4 (3–5) at 3 years of postoperative follow-up. There were 20 recurrences (28.2%). The main postoperative complications were postoperative local pain and compensatory hyperhidrosis. No other adverse events or deaths were observed.

**Conclusion:**

RF-TC for CH could be a feasible, effective, and safe treatment option to improve patients' symptoms of cold sensation. Yet, more researches are needed to verify this potentially efficient and standardized treatment.

## Introduction

Cold hypersensitivity (CH) is a feeling of coldness in the limbs and (or) body of a patient in an environment that is not considered cold by an unaffected person or a strong feeling of coldness at relatively low temperatures mostly seen in Asian women especially those with a low body mass index (BMI) (1, 2). However, the prevalence of this condition in Westerners has not been reported epidemiologically. Some studies have reported that CH is associated with chronic heart failure, diabetes mellitus, dyslipidemia, degenerative arthritis, chronic gastric disease, and chronic rhinitis (3). The main clinical characteristic of patients with cold sensation is an unexplained coldness in the extremities, which is persistent and often requires heavy clothing to relieve symptoms. Some patients with cold sensations have been reported to exhibit other symptoms such as shoulder stiffness, constipation, back pain, fatigue, hot flashes, and generalized pain (4). These discomforts can seriously affect the daily and social life of the patient, thereby causing physical and mental exhaustion and great inconvenience (5, 6).

The onset of CH may be related to the reduced blood supply to the extremities due to excessive vasoconstriction of the extremities (7). Therefore, increasing blood perfusion to the limbs is the key to treating the disease. Currently, the treatment for CH is based on conservative treatments, including lifestyle changes and oral medications, such as warmth, vasodilator drugs, herbs, and capsaicin, which are effective but difficult to maintain in the long term (8–11). Chemical destruction of the lumbar sympathetic nerve using anhydrous ethanol for the treatment of cold sensitization of the lower extremities has been shown to have a more satisfactory effect and can significantly improve the symptoms of coldness in the lower extremities of patients (12). However, effective postoperative maintenance time of patients is generally short and prone to recurrence. Besides, due to the mobility of anhydrous ethanol, it is poorly controllable in clinical application, and the diffused drug may lead to other potential complications (13). Therefore, it is important to explore new treatment strategies for limb cold sensitization disorder to improve patient outcomes and prognosis for clinical guidance.

In recent years, with the rapid development of radiofrequency thermocoagulation (RF-TC), lumbar sympathetic nerve RF-TC has been widely employed in the treatment of neurovascular lesions and local pain syndromes in the lower extremities, and long-term effective symptom relief has been achieved (14–16). Cold hypersensitivity is due to autonomic nerve dysfunction causing somatosensory abnormalities. In this study, we performed radiofrequency thermocoagulation on the sympathetic nerve of the patient to deactivate the lesioned allergic nerve endings and block the conduction pathway of the nerve, thus achieving the purpose of regulating (inhibiting) nerve function. Herein, sympathetic nerve RF-TC was applied to the clinical treatment of patients with CH, and its effectiveness and safety were analyzed to assess postoperative recurrence and patient satisfaction. By analyzing and summarizing the clinical experience, we sought to provide a safe and reliable clinical treatment option for patients with CH.

## Methods

### Subjects

A total of 86 patients diagnosed with CH in the extremities who underwent computed tomography (CT)-guided thoracic (lumbar) sympathetic nerve RF-TC in the pain department of the affiliated Hospital of Jiaxing University from October 2018 to February 2022 were enrolled. The study data were obtained from the hospital's electronic medical record system, eWorldRIS imaging system, etc. In consideration of privacy protection, patient names were excluded from the data analysis process and replaced with the experimental number. Inclusion criteria were: (1) patients with clinically confirmed CH (1); (2) patients whose CH could not remit or whose conservative treatment is ineffective (3) patients who underwent sympathetic RF-TC. Exclusion criteria were: (1) intraoperative injection of anhydrous ethanol to assist neuromodulation; (2) unilateral limb involvement; (3) patients with a disease duration of < 1 month were excluded because transient vegetative dysfunction and the inability to exclude other acute diseases may interfere with the study results. For patients with multiple surgical segments [e.g., 3rd lumbar vertebrae (L3) + 4th thoracic vertebrae (T4)], the final recorded unilateral temperature or perfusion index (PI) values were averaged based on the multiple measurements. In addition, since sympathetic drive may lead to symptoms such as excessive sweating, excessive sweating was included as a concomitant symptom in our study. Because there is no consensus on the surgical segment of sympathetic nerve RF-TC, therefore, based on the experience of our research team, T4 and L3 RF-TC were performed if the symptoms were severe in both palms and both feet and if the symptoms of cold sensation were severe in the lower extremities, L3 radiofrequency was performed as an individualized treatment plan according to the condition located distally, and L2 radiofrequency was performed proximally. This study was approved by the Medical Ethics Committee of the Affiliated Hospital of Jiaxing College (approval number 2022-KY-360).

### Surgical procedure

**Figure 1:**
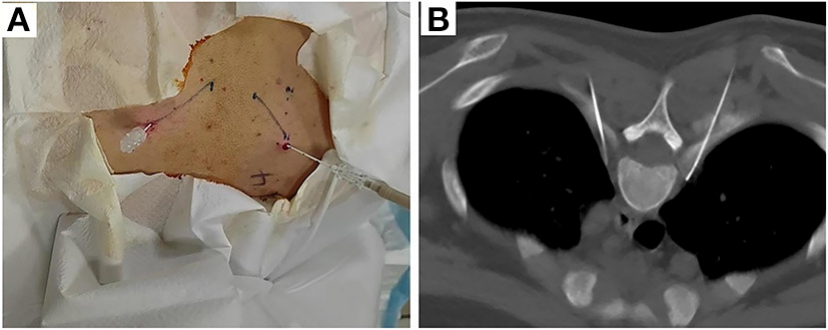
Body surface localization **(A)** and radiofrequency targeting points **(B)** of CT-guided thoracic sympathetic nerve radiofrequency thermocoagulation using the T4 segment as an example.

Thoracic sympathetic nerve RF-TC was performed using the T4 segment as an example. The operation procedure, possible efficacy, and risks of this technique were explained in detail to the patient before surgery, and the patient's informed consent was obtained. After the patient was subjected to preoperative fasting for 6 h, the infusion channel was opened and the patient was placed in a prone position on the CT operating table with the thoracic back exposed and a soft pillow under the chest. The patient's heart rate (HR), non-invasive blood pressure (NIBP), oxygen saturation (SpO2), palmar temperature (T), and peripheral PI (using a Radical-7 monitor from Masimo, USA) were continuously monitored and recorded before and after RF. A localization grid was placed on the skin of the back corresponding to the T4 vertebral body and the intervertebral space (T3-T4) was accurately located using CT localization images. The upper and lower vertebral bodies were scanned with a layer thickness of 3 mm. The target point was the anterior margin of the small head of the rib. Using CT tool software, a straight line was drawn from the target point to the skin through the anterior edge of the 4th rib joint, and the point of intersection with the skin was used as the puncture entry point. The proposed entry depth (distance between the entry point and the target point) and entry angle (angle between the puncture needle and the sagittal plane) were measured. The laser positioning red line of the CT was turned on, the CT bed was moved to the selected puncture level, and the puncture entry points on both sides were marked on the positioning red line using a marker. After routine disinfection and spreading of the towel, the selected puncture site was locally anesthetized with 1% lidocaine, and the needle was inserted under CT guidance according to the proposed angle and depth of insertion: bilaterally with a 10-cm-long, 10-mm bare end 7-gauge RF needle through the T3-T4 intervertebral space to the anterior border of the small head of the T4 rib (Figure 1).

**Figure 2:**
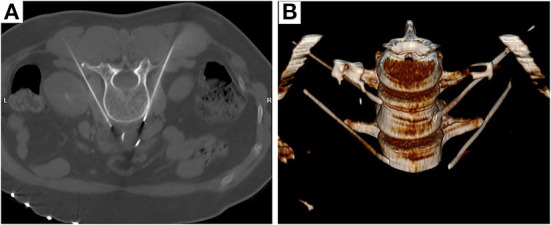
Radiofrequency targeting points **(A)** and three-dimensional reconstruction **(B)** of CT-guided thoracic sympathetic nerve radiofrequency thermocoagulation using the L3 segment as an example.

Lumbar sympathetic RF-TC was performed using the L3 segment as an example. The procedure was performed under CT guidance. After opening the peripheral intravenous fluid, the patient was placed in a prone position on the CT treatment table and a thin pillow was placed under the abdomen to expose the puncture site. The patient was monitored with continuous cardiac monitoring of HR, NIBP, and SpO2, as well as dynamic monitoring of T and PI. A positioning shed was placed next to the lumbar spinous process. L3 was identified on the CT scan, and the puncture level was the level containing bilateral transverse processes. The puncture target was the anterolateral border of the L3 vertebral body and the anteromedial border of the psoas major muscle. A CT tool ruler was used to design the puncture path using the puncture target as the target. The puncture site was marked on the body surface with a marker and local infiltration anesthesia was applied to the puncture site with lidocaine. A 15-cm 22G RF puncture needle (effective tip 10 mm) was slowly advanced according to the set puncture path to reach the target point (between the anterolateral border of the L3 vertebral body and the anteromedial border of the psoas major muscle) (Figure 2).

After the puncture was completed, the needle core was withdrawn and the RF electrode was inserted along the trocar after back pumping free of fluid and gas. Sensory testing at 50 Hz and 0.2-1.0 mA and motor testing at 2 Hz and 1.0 mA max were performed through the radiofrequency machine. Confirmation of the absence of muscle twitching in the area of spinal nerve innervation indicated the feasibility of RF treatment. The radiofrequency temperature and time were adjusted to 95°C and 300 s, respectively, for two cycles of RF-TC. The procedure was considered successful when the temperature of both hands increased by >2°C or the PI increased by >50% of the baseline value and the palms of both hands or feet changed from wet cold to dry heat. For patients without significant postoperative complications, the puncture site was covered using an appropriate sterile dressing, and patients were returned to the ward.

### Endpoint

The primary observational endpoint of this study was the Visual Analog Scale (VAS) score at each postoperative time point. Based on the VAS scores of 0 to 10 for pain, patients were classified as having no cold sensation (0 scores), mild cold sensation (1–3 scores), moderate cold sensation (4–6 scores), and severe cold sensation (7–10 scores). Secondary observational endpoints included postoperative temperature and PI of both hands or feet, Pittsburgh Sleep Quality Index (PSQI) scores at each postoperative time interval, recurrence rate, the incidence of postoperative pain, incidence of compensatory hyperhidrosis, and patient satisfaction. The PSQI is used to assess the quality of sleep of patients – with scores ranging from 0 to 21 – with lower scores indicating better sleep quality. In the dynamic assessment of VAS and PSQI scores, data were collected based on the time of the patient's surgery as follows: during the preoperative period (t0), immediate postoperative period (t1), 1 month (t2), 3 months (t3), 6 months (t4), 1 year (t5), 2 years (t6), and 3 years (t7). Subjects who met the follow-up requirements were counted. Missing data due to missed visits were excluded in the corresponding analysis.

### Statistical analyses

Data were statistically analyzed using SPSS 26.0 (SPSS lnc, Chicago, IL) and R software. The Shapiro-Wilk test was used to test the normality of the data and normally distributed data were expressed as the mean and standard deviation (mean ± SD) and compared using a *t*-test. Non-normally distributed data were expressed as median (interquartile spacing) and the Friedman test was used for variance analysis. The Bonferroni test with corrected significance level was used for multiple comparisons. Qualitative data were expressed as frequencies (%) and compared using the chi-square test. The R software was used to draw violin plots showing the changes in VAS and PSQI scores between preoperative and postoperative time points in 28 patients with a one-way repeated ANOVA. *P* < 0.05 was considered statistically significant.

## Results

### Patients characteristics

**Figure 3:**
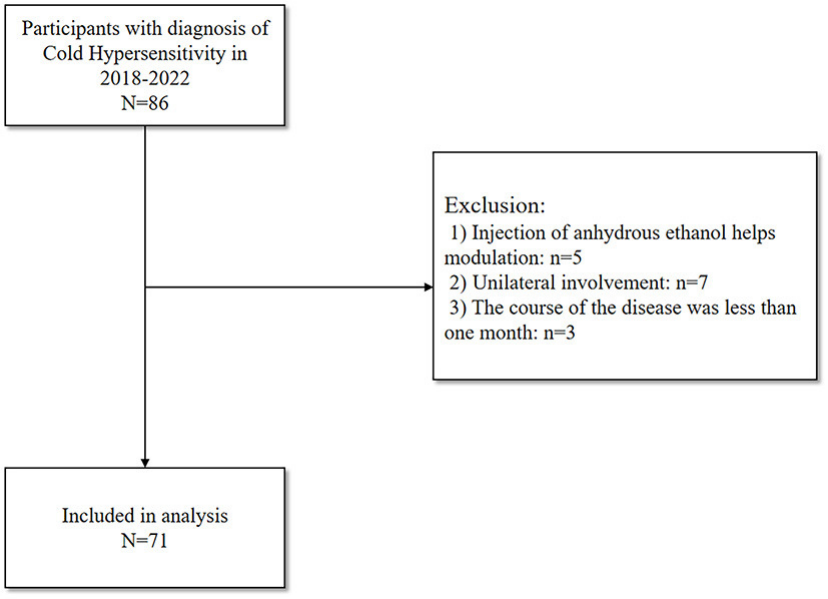
Flowchart depicting the detailed process of patients' enrollment.

A total of 86 patients were recruited, of which 15 were excluded and 71 were entered into the final analysis. The flow chart of patient enrollment is shown in Figure 3. Preoperative baseline characteristics of patients, including gender, age, height, weight, BMI, disease duration, lesion site, whether combined with hyperhidrosis, surgical segment, extremity temperature, peripheral PI, VAS score and PSQI score (Table 1). The Table 1 also shows that the patients with cold sensitization in this study were mostly middle-aged women and the lesions were mostly in the lower extremities.

**Table 1:** Characteristics of participants.

**Characteristics**	**Statistic**
Gender (*n*, %)	
Male	27(38.0)
Female	44(62.0)
Age (year, mean ± sd)	54.65 ± 11.71
Height (cm, IQR)	160(156,169)
Weight (kg, mean ± sd)	59.41 ± 9.83
BMI (kg/m^2^, mean ± sd)	22.59 ± 3.26
Course of disease (month, IQR)	60(24,96)
Position (*n*, %)	
Upper limbs	1(1.4)
Lower limbs	47(66.2)
Limbs	23(32.4)
Hyperhydrosis (*n*, %)	
No	61(85.9)
Yes	10(14.1)
Segments of surgery (*n*, %)	
T4	3(4.2)
L2	2(2.8)
L3	37(52.1)
L3+T3	4(5.6)
L3+T4	11(15.5)
L2+L3	9(12.7)
L2+L3+T3	1(1.4)
L2+L3+T4	4(5.6)
Temperature (°C, mean ± sd)	
Left	29.51 ± 1.83
Right	29.49 ± 1.85
PI (IQR)	
Left	1.10(0.84,1.51)
Right	1.22(0.75,1.85)
VAS (IQR)	6.00(6.00,7.00)
PSQI (IQR)	13.00(11.00,14.50)

BMI, body mass index; PI, perfusion index; VAS, visual analogue scale; PSQI, pittsburgh sleep quality index.

### VAS and PSQI scores

**Figure 4:**
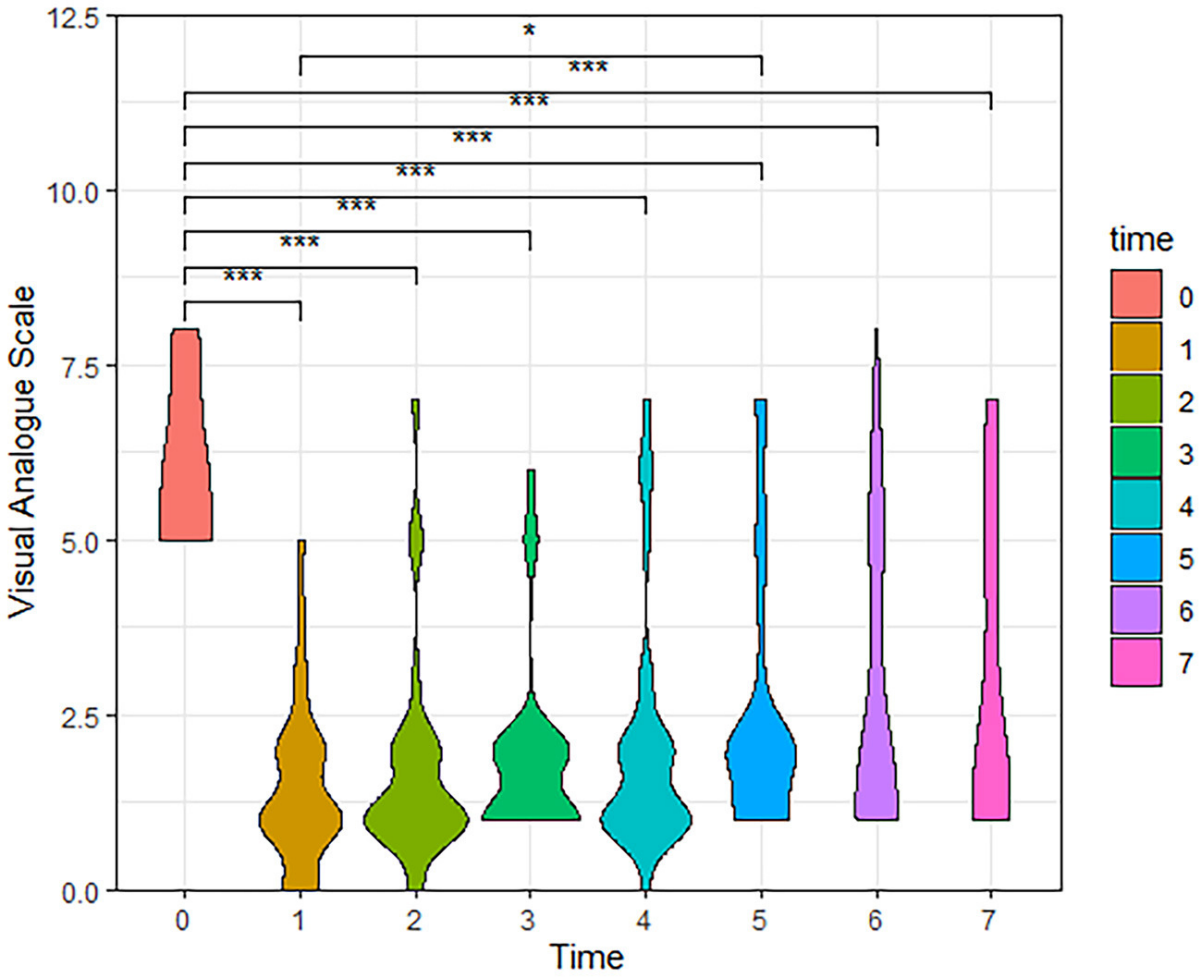
The Violin Plot of Visual Analog Scale (VAS) scores based on 28 patients followed up to 3 years postoperatively. Compared with the preoperative, patients had significantly lower VAS scores at all postoperative time periods. (t0, Pre-operation; t1, Post-operation; t2, One month; t3, Three months; t4, Six months; t5, One year; t6, Two years; t7, Three years, ***P < 0.001, *P < 0.05).

**Figure 5:**
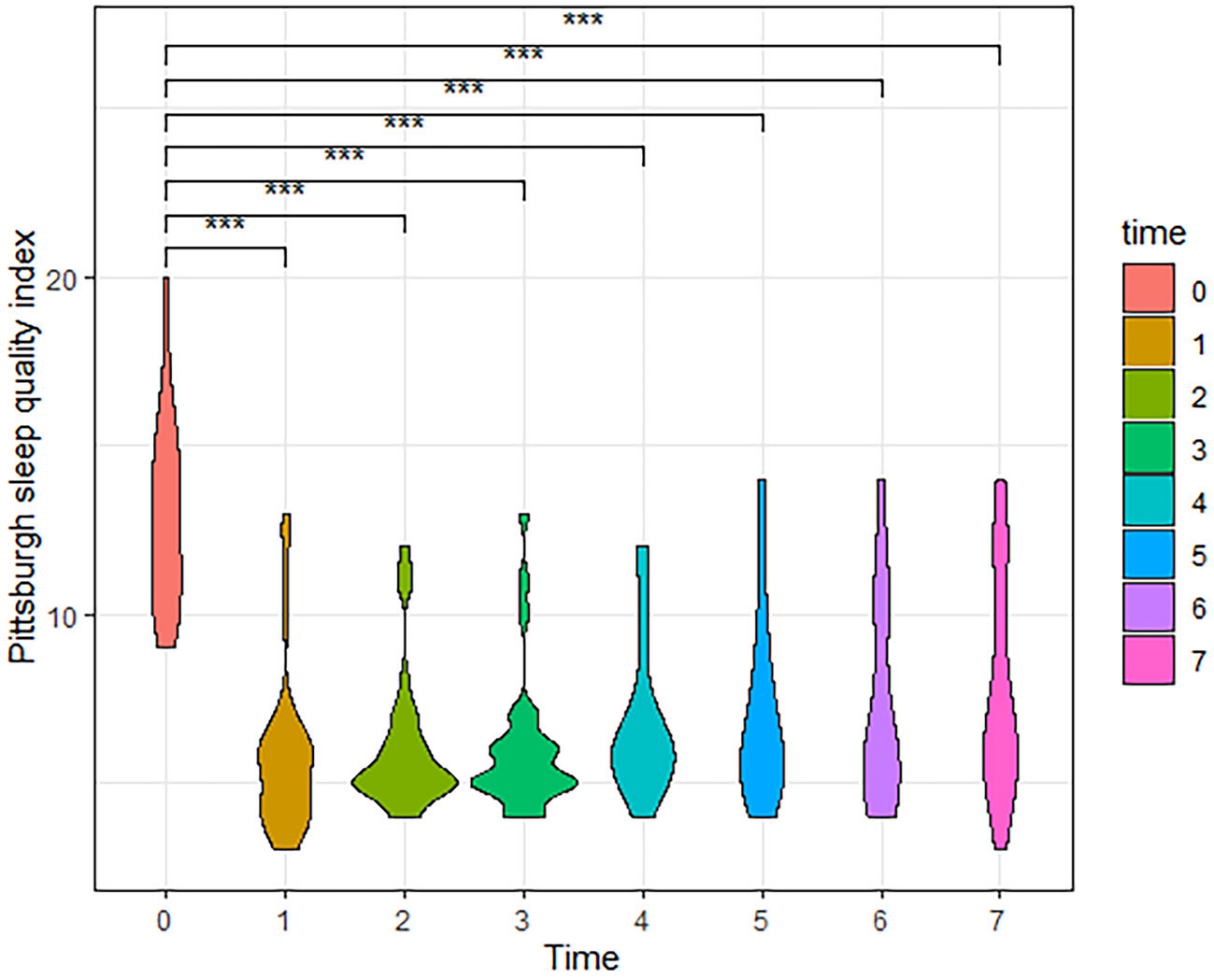
The Violin Plot of Pittsburgh Sleep Quality Index (PSQI) scores based on 28 patients followed up to 3 years postoperatively. Compared with the preoperative, patients had significantly lower PSQI scores at all postoperative time periods. (t0, Pre-operation; t1, Post-operation; t2, One month; t3, Three months; t4, Six months; t5, One year; t6, Two years; t7, Three years, ***P < 0.001).

According to the follow-up results, the number of cases in our final analysis varied by time period: 71 cases for the t0-t4 time period, 58 cases for t5, 42 cases for t6, and 28 cases for t7. Analysis showed that both VAS and PSQI scores decreased significantly in the patient t1-t7 group compared to t0. As shown in Table 2, patients had a moderate level of cold sensation (median 6 points) preoperatively, which decreased to a mild level (median 1 or 2 points) at all postoperative time points. In addition to this, the table also shows that the patients' VAS scores rebounded after 6 months postoperatively. Meanwhile, violin plots were drawn based on 28 patients who were followed up for 3 years postoperatively. The results showed that VAS and PSQI scores decreased significantly in all postoperative time periods compared with the preoperative period (*P* < 0.001, Figures 4, 5).

**Table 2:** Visual Analogue Scale (VAS) and Pittsburgh sleep quality index (PSQI) of participants at different time interval (IQR).

**Time interval**	**Number of participants**	**Visual analogue scale**	**Pittsburgh sleep quality index**
t0	71	6.00(6.00–7.00)	13.00(11.00–14.50)
t1	71	1.00(1.00–2.00)	6.00(4.00–6.00)
t2	71	1.00(1.00–2.00)	5.00(5.00–7.00)
t3	71	1.00(1.00–2.50)	6.00(5.00–7.00)
t4	71	1.00(1.00–3.00)	6.00(5.00–8.00)
t5	58	2.00(1.00–4.00)	6.00(5.00–8.00)
t6	42	2.00(1.00–5.00)	6.00(5.00–10.00)
t7	28	2.00(1.00–5.00)	6.00(5.00–10.25)

t0, Pre–operation; t1, Post–operation; t2, One month; t3, Three months; t4, Six months; t5, One year; t6, Two years; t7, Three years.

### Temperature and PI

As shown in Table 3, the patient's preoperative peripheral temperature was 29.51°C on the left side and increased to 32.50°C postoperatively; the right side temperature was 29.49°C preoperatively and 32.8°C postoperatively. The PI of the left and right sides before surgery was 1.10 and 1.22 respectively, and increased to 6.38 and 7.22 after conditioning respectively. In short, compared with the preoperative period, the patient's postoperative terminal temperature and PI of the left and right sides were significantly higher, and the difference was statistically significant (*P* < 0.001).

**Table 3:** Comparison temperature and perfusion index before and after modulation (mean ± sd or IQR).

**Variables**	**Temperature (** **°** **C)**		**Perfusion index**	
	**Left**	**Right**	**Left**	**Right**
Pre–operation	29.51 ± 1.83	29.49 ± 1.85	1.10(0.84–1.51)	1.22(0.75–1.85)
Post–operation	32.50(31.00–34.25)	32.80(30.85–33.85)	6.38 ± 3.38	7.22 ± 3.70
*P*–value	<0.001	<0.001	<0.001	<0.001

### Postoperative complications and satisfaction

At follow-up until 3 years after surgery, 20 (28.2%) recurrences, 48 (67.6%) non-recurrences, and 3 (4.2%) lost visits were documented. The main postoperative complications were postoperative local pain and compensatory hyperhidrosis. Postoperative local pain was observed in 7 (9.9%) patients, of whom 5 had pain at the puncture site and 2 had pain in the groin area. The pain was gradually relieved in all patients. Seventeen (23.9%) patients had postoperative compensatory hyperhidrosis, which mainly manifested as increased sweating on the chest and back. Postoperative perineal edema, burning sensation in the eyes, and absence of sweating in both feet were observed in one case each; these complications resolved completely over time in all patients. No other adverse events or deaths were observed. The overall postoperative patient satisfaction score was 4 (3–5) (Table 4).

**Table 4:** Complications and satisfaction of participants after modulation.

**Variables**	**Statistics**
Relapse*(n, %)*	
No	48(67.6)
Yes	20(28.2)
Not follow–up	3(4.2)
Post–operative pain*(n, %)*	7(9.9)
Compensatory hyperhidrosis*(n, %)*	17(23.9)
Satisfaction (IQR)	4(3–5)

Satisfaction: scores from 1 to 5, the higher the score, the more satisfied the patient is.

## Discussion

CH is a unique pathological condition. Patients have an abnormal subjective sensitivity to cold in the back, extremities, other localized areas of the body, or the whole body, despite high ambient temperatures. For most patients, this cold sensation lasts for years and severely disrupts their daily life (4). An epidemiological study involving 2201 people in Korea showed that up to one-third of the subjects developed cold sensations in the order of the feet, abdomen, and hands, with 17.9% of the subjects accounting for both hands and feet and 12.3% for all three sites (17).

Although the exact mechanism of CH remains unclear, it has been suggested that its pathogenesis is associated with a hypersensitive vasoconstrictor response and a genetic configuration and imbalance of autonomic nerves, leading to impaired peripheral circulation (2, 18). It has also been suggested that CH may be highly associated with sympathetic hyperfunction, with excessive myelin formation and an increase in the proportion of nerve fibers thought to contribute to increased sympathetic excitability (19). From the perspective of conventional medicine, cold sensitization cannot be completely distinguished from Raynaud's disease but it can be diagnosed by observing changes in the skin tone of the hands or feet in a cold environment, a view that considers cold sensitization as latent Raynaud's disease (20, 21). Therefore, the current pharmacological treatment for cold sensation is still based on vasodilators or antihypertensive drugs (22). However, long-term medication can lead to some serious drug side effects, such as dizziness and headache and cardiovascular, hepatic, and renal impairment, and the overall efficacy is somewhat unsatisfactory. Moreover, there are no effective interventions that can improve patients' CH symptoms in the long term.

Radiofrequency thermal coagulation generates a temperature of over 45°C by outputting a constant high-frequency current, which causes ion movement and heat generation in the target tissue, resulting in ablative thermal coagulation of the nerve (23). In 1948, percutaneous thoracic segment sympathetic nerve RF-TC was first reported, and it was suggested that this technique has the advantages of being safe, effective, simple to operate, repeatable, quick recovery, well-tolerated, etc (24). Therefore, it can be used in clinical applications. With the continuous research on RF-TC and sympathetic nerve function, sympathetic nerve RF-TC has been widely used in Raynaud's disease, hyperhidrosis, and neurogenic pain, with remarkable efficacy and no serious complications (25–27).

Sympathetic nerve RF-TC is a minimally invasive percutaneous procedure with the advantages of less trauma, lower cost, and fewer complications compared with open or lumpectomy-assisted sympathectomy and chemical nerve release (28). In the present study, the optimal puncture path was designed under CT guidance, and the needle was inserted according to the pre-measured depth and angle, which reduced the number of punctures to a certain extent and improved the success rate of the puncture. In addition, local infiltration anesthesia before puncture and intravenous administration of an appropriate amount of analgesic before radiofrequency treatment significantly reduced the intraoperative pain of patients and improved their treatment satisfaction. It provided patients with a comfortable, convenient, and humanized medical experience, which helped improve the quality of perioperative patient care and promote recovery.

PI is the ratio of pulsatile blood flow to non-pulsatile blood flow (other tissues) at the monitoring site and reflects the quantitative value of real-time changes in blood flow around the monitoring site (29). The ratio increases with an increase in the blood flow through the detector site because the non-pulsatile component does not change. Previous studies have shown that PI is more sensitive and specific than skin temperature changes and can be used as an indicator of successful sympathetic modulation (30). In our study, sympathetic RF-TC was used to treat CH, and a comparison of preoperative and postoperative controls revealed that T and PI indices were significantly higher immediately after treatment compared to the preoperative period. This demonstrated the efficiency of our approach.

Different sympathetic nerve segments are selected based on the location of the lesion to provide targeted and individualized treatment for patients with cold sensitization. For example, T3 segment radiofrequency is used for head and face coldness, T4 for upper limbs or chest and back, L2 for the lumbar abdomen, L3 for the proximal lower extremities, and L4 for the distal lower extremities. In case of generalized or multiple sites of chills, combined multi-segment radiofrequency treatment was performed. Our data showed that VAS and PSQI scores were significantly lower in all postoperative time periods compared with the preoperative period. In addition, 28 patients were followed up for 3 years and found that the preoperative VAS and PSQI scores were lower at all time points than preoperatively, and the differences were statistically significant. The preoperative degree of cold sensation in the patients was mainly moderate and severe, which seriously affected their daily life and sleep. After a radiofrequency treatment, the patients were followed up regularly for a long period, and the median cold sensation degree scores of the patients were mild at all postoperative time points. The sleep quality of patients was greatly improved. Accordingly, it was inferred that sympathetic RF-TC for CH can achieve good long-term results.

To make the degree of nerve destruction more complete and thorough, and based on the fact that the extent of destruction at the exposed end of the radiofrequency needle tip expands with the increase in temperature, a high-temperature (95°C) and long-duration (300 s) RF-TC was chosen for the treatment of CH in this study. A follow-up of up to 3 years after surgery revealed that 20 patients (28.2%) had a recurrence. This may be due to the incomplete destruction of nerve fibers and regeneration of nerve fibers after RF-TC. Although neurons cannot be regenerated or their growth is slow, nerve fibers can be regenerated (31). Therefore, some patients may still relapse after RF-TC treatment.

Postoperative complications are of particular concern to both physicians and patients. The main postoperative complications were postoperative local pain and compensatory hyperhidrosis. Postoperative local pain manifested as pain at the puncture site, and the degree of this pain was usually mild. The pain was relieved in all patients within 1 month after routine administration of non-steroidal anti-inflammatory drugs. Compensatory hyperhidrosis was due to the severance of sympathetic nerves innervating the sweat glands of the hand or foot after sympathetic radiofrequency, allowing functional compensation of sweat glands in another part of the body to maintain a state of balance between normal body temperature and sweating. Furthermore, compensatory hyperhidrosis after sympathetic nerve RF-TC was mainly manifested by increased sweating in the thoracic back or lumbar abdomen. Previous studies found that compensatory hyperhidrosis occurred in approximately 3-98% of patients after sympathetic nerve chain dissection, with the vast majority of patients having mild to moderate hyperhidrosis (32). It was confirmed through our follow-up that this level of compensatory sweating was acceptable. We also identified one case each of perineal edema, burning sensation in the eyes, and an absence of sweating in both feet. This was a complication of the innervated areas due to the destruction of thoracic or lumbar sympathetic nerves by RF-TC. However, all symptoms resolved completely over time. Moreover, no other serious adverse events or deaths were observed.

This study has several limitations. Firstly, this study is a single-center, retrospective study with small sample size and few observed indicators, which is subject to some bias; thus, large-scale, multicenter, prospective studies are needed to verify our results. Secondly, only the fingertip T and PI of patients before and immediately after treatment were collected, and changes in pedal T and PI of patients were not monitored during the follow-up, which lacked some objective basis. Thirdly, the number of follow-up cases after 1 year postoperatively gradually decreased, which might have impacted our results. Finally, the VAS scores of patients during the follow-up period may be biased due to variations in ambient temperature, season, and patient's mood at the time.

## Conclusions

In summary, sympathetic RF-TC for CH may be an effective, minimally invasive, safe and reliable treatment option for long-lasting relief of cold symptoms in patients with CH. However, the precise indications and clinical implications of sympathetic RF-TC in the treatment of CH warrant further exploration, and more studies are needed in the future to validate this potentially effective and standardized treatment.

## Data availability statement

The original contributions presented in the study are included in the article/supplementary material, further inquiries can be directed to the corresponding authors.

## Ethics statement

The studies involving human participants were reviewed and approved by the Medical Ethics Committee of the Affiliated Hospital of Jiaxing College. The patients/participants provided their written informed consent to participate in this study. Written informed consent was obtained from the individual(s) for the publication of any potentially identifiable images or data included in this article.

## Author contributions

Study concept, design, and revision of the manuscript: YL, ZZ, and CN. Drafting of the manuscript: YL, CX, ZZ, and CN. Follow up and data collection: CX, JX, and YL. Implement the trial: HN and ZZ. Analysis and interpretation of data: CX, HN, and ZZ. All authors have read and approved the final manuscript.

## Conflict of interest

The authors declare that the research was conducted in the absence of any commercial or financial relationships that could be construed as a potential conflict of interest.

## Publisher's note

All claims expressed in this article are solely those of the authors and do not necessarily represent those of their affiliated organizations, or those of the publisher, the editors and the reviewers. Any product that may be evaluated in this article, or claim that may be made by its manufacturer, is not guaranteed or endorsed by the publisher.
